# Being a family medicine resident in the United States

**DOI:** 10.1002/jgf2.392

**Published:** 2020-10-26

**Authors:** Kaku Kuroda, Ryuichi Ohta, R. Eugene Bailey

**Affiliations:** ^1^ Department of Family Medicine SUNY Upstate Medical University Syracuse NY USA; ^2^ Community Care Unnan City Hospital Shimane Japan

To the Editor,

I read previous qualitative studies that mentioned that the difficulty of English communication is the greatest challenge for Japanese international medical graduates (IMG) who receive US clinical training.^1^, ^2^ I strongly agree with these extracted concepts, from my experience in the family medicine residency in Syracuse, New York. I realized how challenging this cultural shift would prove to be. However, is it worth struggling with the language barrier to complete family medicine residency even though we have an established medical education in our own language? The answer is undoubtedly “Yes.” I would like to add three standpoints.

First, diversity in the United States makes Japanese physicians more aware of the importance of the social determinants of health (SDH). Understanding SDH is valued the most for effective care in family medicine.^3^ For example, I saw many pregnant women who used illicit drugs. Their backgrounds were diverse in terms of race, immigration history, religion, access to firearms, and socioeconomic status. Many of them requested to go home even on the day they had delivered the baby or underwent surgery because of concerns about health insurance coverage.

Second, working in the United States allowed Japanese physicians to overcome the biggest obstacle in learning evidence‐based medicine (EBM), which is English proficiency.^4^ Everyday EBM discussions in English can never be truly experienced in Japan. Once I stopped translating the English articles for EBM discussion, the speed and efficiency of researching clinical questions increased drastically.

Third, comprehensive learning regarding patient care in American family medicine residency can facilitate Japanese physicians to learn the continuity and comprehensiveness of patient care. Japan lacks generalist training historically compared to the United States, and many Japanese physicians who had US clinical training have reformed the generalist field in Japan.^5^ During residency, residents have longitudinal ambulatory practice once a week throughout the year to focus only on family medicine. In addition, we practice the full scope of family practice, including maternity care.

In my case, 8 years had passed since preparing to become a resident in the United States, including passing the US medical license examination, having clinical experience in US Naval Hospital Okinawa, and completing a time‐consuming matching process, as a previous study showed.^1^ Once the residency began in July, I always felt miserable and guilty because of my insufficient English skills and different discussion attitudes. Most of my energy was consumed in avoiding miscommunication. There were a number of confusing ways that native English speakers applied seemingly endless nuances.^2^

However, I confirm that Japanese residents can be diligent and determined to perform at a higher level in overcoming the language barrier, as former Japanese residents told me. I believe that this experience allows us to deepen our insight into how family medicine can be applied to the Japanese medical system in the best context after completing residency in two languages and cultures from a global perspective. Thus, I assert that the experience of Japanese IMG is definitely worthwhile to face tremendous challenges.

## CONFLICT OF INTEREST

The authors have stated explicitly that there are no conflicts of interest in connection with this article.
